# Tumor immunity landscape in non-small cell lung cancer

**DOI:** 10.7717/peerj.4546

**Published:** 2018-03-23

**Authors:** Xiaoqing Yu, Xuefeng Wang

**Affiliations:** Department of Biostatistics and Bioinformatics, H. Lee Moffitt Cancer Center & Research Institute, Tampa, FL, USA

**Keywords:** Tumor immunity, Mutational signature, Non-small cell lung cancer, Cytolytic activity, TCR clonality, Immunotherapy

## Abstract

Even with the great advances in immunotherapy in recent years, the response rate to immune checkpoint inhibitor therapy for non-small cell lung cancer is only about 20%. We aimed to identify new features that would better predict which patients can benefit from an immune checkpoint blocker. This study is based on the publicly available gene expression data from The Cancer Genome Atlas lung cancer samples and the newly released mutation annotation data. We performed a comprehensive analysis by correlating patient cytolytic activity index, mutational signatures, and other immune characteristics in four stratified patient groups. The results cytolytic activity index are highly correlated with immune infiltration scores, T cell infiltration scores and TCR clonality scores in lung cancer. In addition, we observed that the mutational event signatures might play a more important role in predicting immunotherapy response in squamous cell carcinoma and two subgroups of adenocarcinomas. Our analysis illustrates the utility of integrating both tumor immune and genomic landscape for a better understanding of immune response in lung cancer.

## Introduction

Non-small cell lung cancer (NSCLC) accounts for more than 85% of incidences of lung cancers. Even with the great advances in immunotherapy in recent years, the response rate to immune checkpoint inhibitor therapy for NSCLC, such as anti-PD1/PDL1 therapy, is only about 20%. Efforts are now underway to identify biomarkers that predict which patients benefit from immune checkpoint blocker, as well as reasons why patients don’t respond to the current treatment. A key concept in cancer immunotherapy is that cancer malignant cells, which would normally be recognized by T cells, can develop mechanisms to evade the human immune system by disrupting checkpoint molecules. The goal of effective immunotherapy is thus to restore the ability of the immune system to detect and attack cancer cells. A better understanding of how tumor cells and immune cells interact and contribute to the tumor microenvironment is crucial in improving the efficacy of immunotherapy and in developing alternative treatments. Tumor cells express antigens that can be specifically recognized by immune cells such as T cells and B cells. On the other hand, cancer researchers are interested in whether there is sufficient amount of infiltrating lymphocytes in tumor tissues, their cell type distribution, and what proportion of these immune cells are activated and functional. Preliminary studies (Schumacher & Schreiber, 2015; Snyder et al., 2014) suggest that tumors with high somatic mutation burden which is associated with high neoantigen load are more likely to respond to immune checkpoint inhibition. However, the association between mutational and neoantigen load and clinical outcomes did not reach statistical significance in many current studies, partly due to a still limited number of patient cases. Additionally, mutation burden (Rizvi et al., 2015) combined with high expression of PD-L1 expression is also a potential predictor of response in NSCLS. The presence of tumor-infiltrating lymphocytes (TILs) has also been recognized as a favourable prognostic biomarker across a wide range of cancers. High TIL proportion with elevated TCR clonality is usually associated with increased efficacy of checkpoint blockade immunotherapy. Based on RNA-seq samples in TCGA, (Li et al., 2016a; Li et al., 2016b) has successfully inferred the proportions of immune cell types and complementarity-determining region 3 (CDR3) sequences of tumor-infiltrating T cells in across 29 cancer types. All these findings and tools have greatly facilitated high-throughput immunity profiling and in-silico discovery of novel immunotherapy  targets.

Several recent studies indicate that cytolytic activity plays an important role in the immunoregulatory processes that influence cancer development and therapy response. Based on large-scale genomic datasets of solid tumor, Rooney et al. (2015) showed that the gene signature of cytolytic effector activity, based on the transcript levels of the granzyme A (GZMA) and perforin (PRF1), had an association with neoantign load and suggested potential new mechanisms that enable tumors to resist immune attack. A recent pan-cancer immunogenomic analyses (Charoentong et al., 2017) also reveals that the immunogenicity of the tumor can be represented by the cytolytic activity, and which can be a predictor of response to checkpoint blockade in melanoma. From melanoma patients treated with the anti-CTLA4 antibody ipilimumab, Patel et al. (2017) further found that intratumoral cytolytic activity was strongly correlated with CD8 + T cell infiltration in the tumors and with the expression of the MHC class I antigen processing pathway. However, it is unknown whether the stratification based on cytolytic activity, combined with mutation load and other tumor infiltrating indicators, may reveal new tumor subtypes or patient treatment groups in NSCLS.

To better characterize the relationship among immunogenicity, tumor mutational signature and immune infiltrates in lung cancer, we analyzed all the available molecular data in TCGA based on existing and newly developed bioinformatics tools. We carried out the comprehensive immunogenomic characterization of tumor samples in Lung Adenocarcinoma (TCGA-LUAD) and Lung Squamous Cell Carcinoma (TCGA-LUSC). This analysis was especially timely, given that the full mutation annotation data for LUSC samples was not publicly available until very recently (released by GDC portal in late 2017). Additionally, we investigated the relationships of immune signatures and clinical outcomes, as well as germline mutations among patients with lung cancer. Through correlating all the derived immune, genomic and clinical metrics, we address several questions: how cytolytic activity in lung cancer correlate with mutation load; do the patient stratification analyses based on cytolytic activity and tumor mutational signatures reflect different mechanisms of immune evasion; and whether we can develop a new set of immunogenecity scores, which serve as a more accurate predictor of response to immune checkpoint inhibitor therapy with lung cancers.

## Methods

### Data

Molecular data from The Cancer Genome Atlas for Lung Adenocarcinoma (LUAD) and Lung Squamous Cell Carcinoma (LUSC) was downloaded from the cBio Cancer Genomics Portal (http://www.cbioportal.org/), Broad Firehose website (https://gdac.broadinstitute.org/), and from Genomic Data Commons Data Portal (https://portal.gdc.cancer.gov/). A total of 520 LUAD and 504 LUSC patient samples were used to study the immune and genomic signatures (Table S1). In this cohort, 515 LUAD and 492 LUSC samples have matched somatic mutation and RNA-seq data. Clinical data for these patients was obtained from cBio portal.

### Mutation analysis

Somatic mutation analysis of LUAD and LUSC tumor samples was based on whole exome sequencing (WES) data. We extracted all the identified mutations and their basic information from the publicly available WES mutation annotation format (MAF) files. R package deconstructSigs (version 1.8.0) was employed to infer the contribution of known mutational signatures to the observed mutation profile within each tumor sample (Rosenthal et al., 2016). We considered the 30 mutational signatures cataloged at COSMIC (http://cancer.sanger.ac.uk/cosmic/signatures) (Alexandrov et al., 2015). First, the frequency of mutations observed in the 96 tri-nucleotide contexts was calculated for each sample using the “mut.to.sigs.input” function. Then, weights of the 30 reference mutational signatures were estimated using “whichSignatures” function, with trinucleotide’s occurence normalized from exome to genome level. Based on the initial analysis provided at COSMIC, there are seven major mutational signatures presented in LUAD samples and five major signatures in LUSC, which are possibly associated with smoking, age, APOBEC, etc. To explore the potential role of germline variants in lung cancer immunity development, we acquired SNPs genotyped in TCGA with the Affymetrix SNP Array 6.0 from the GDC Legacy Archive portal (https://portal.gdc.cancer.gov/legacy-archive).

### Immune profiling based on gene expression

Immune cytolytic activity (CYT) of LUAD and LUSC sample was measured by taking the geometric mean of expression values from two markers GZMA and PRF1 (Rooney et al., 2015). The gene expression values of tumor samples were calculated based on log2 transformed RSEM (RNA-Seq by Expectation Maximization) values, which are normalized counts provided in broad Firehose portal. CIBERSORT (Newman et al., 2015) algorithm was used to deconvolve immune cell infiltrates in tumor based on RNA-seq gene expression values. We calculated the relative frequency of 22 tumor-infiltrating immune cell subsets in all TCGA lung cancer samples, based on the CIBERSORT default parameters. Additionally, we applied a more recently developed method (Li et al., 2016b), which only estimates frequencies of six major immune cell subtypes but with an improved algorithm geared towards RNA-seq data. We also compare the above results with the tumor-infiltrating T cell repertoire (TCR) profiles of lung cancer samples, which were inferred based on de novo assembly of complementarity determining region 3 (CDR3) sequences from paired-end RNA-seq (Li et al., 2016a). Finally, the score for the relative tumor infiltration levels of 24 immune cell types from all available lung cancer samples were calculated based on a method called single sample gene set enrichment analysis ssGSEA (Şenbabaoğlu et al., 2016; Subramanian et al., 2005). Three immune infiltration scores of TCGA lung cancer patients provided from (Şenbabaoğlu et al., 2016) were also included in this analysis, which are the overall immune infiltration score (IIS), the T cell infiltration score (TIS) and the antigen presenting machinery (APM) score.

### Patient stratification analysis based on CYT and mutation burden

Based on the calculated immune cytolytic activity status and mutation load, we select a subset of patients (for each of LUAD and LUSC cohort) with extreme CYT score and mutation load values. These patients are classified into four groups: I, referring to high mutation load and high CYT, II, referring to high mutation load and low CYT, III, referring to low mutation load and high CYT, and IV, referring low mutation load and low CYT. We then compare their mutation spectrum, immune profiles, as well as clinical outcomes. The cut-off values of CYT and mutation load defined the four groups, as well as TCGA sample IDs, are listed in the Supplementary Information.

## Results

The mutation load, i.e., total number of mutation (log scale), calculated from the new WES data (GDC late 2017 version) data. The median and range of mutation load values (when converted) are consistent with the estimates of somatic mutation prevalence (number of mutations per megabase) reported in previous studies (Alexandrov et al., 2013), in which lung cancer samples were ranked second highest in all cancers after melanoma. The median mutation load of LUSC patients is slightly higher than LUAD but with smaller variation. The median CYT values of LUAD and LUSC samples are 7.514 and 7.374, respectively. According to previous analysis (Rooney et al., 2015), the CYT values for lung cancer is among the highest in all TCGA cancer types, only second to Kidney Renal Clear Cell Carcinoma. The scatterplots of CYT and mutation load as shown in the Fig. S1, however, indicate that there is no significant correlation (*r* = 0.03 and 0.08 for LUAD and LUSC samples, respectively) or pattern between the two metrics in lung cancer patients. As such, we selected 100 patients who presented with extreme CYT and mutation load values from the two TCGA lung cancer cohorts for the further stratification analyses. These patients are evenly distributed among four subgroups defined previously, i.e., there are 25 patients in each subgroup. The resulted cutoff levels of CYT and mutation load in each group are listed in the Fig. S1. These cutoff values were set to select patients with top or bottom of CYT/mutation or their combination in each subgroup. Analysis of the overall survival did not reveal significant difference among these groups. The exact TCGA lung samples assigned to these groups are noted in the Table S1.

**Figure 1:**
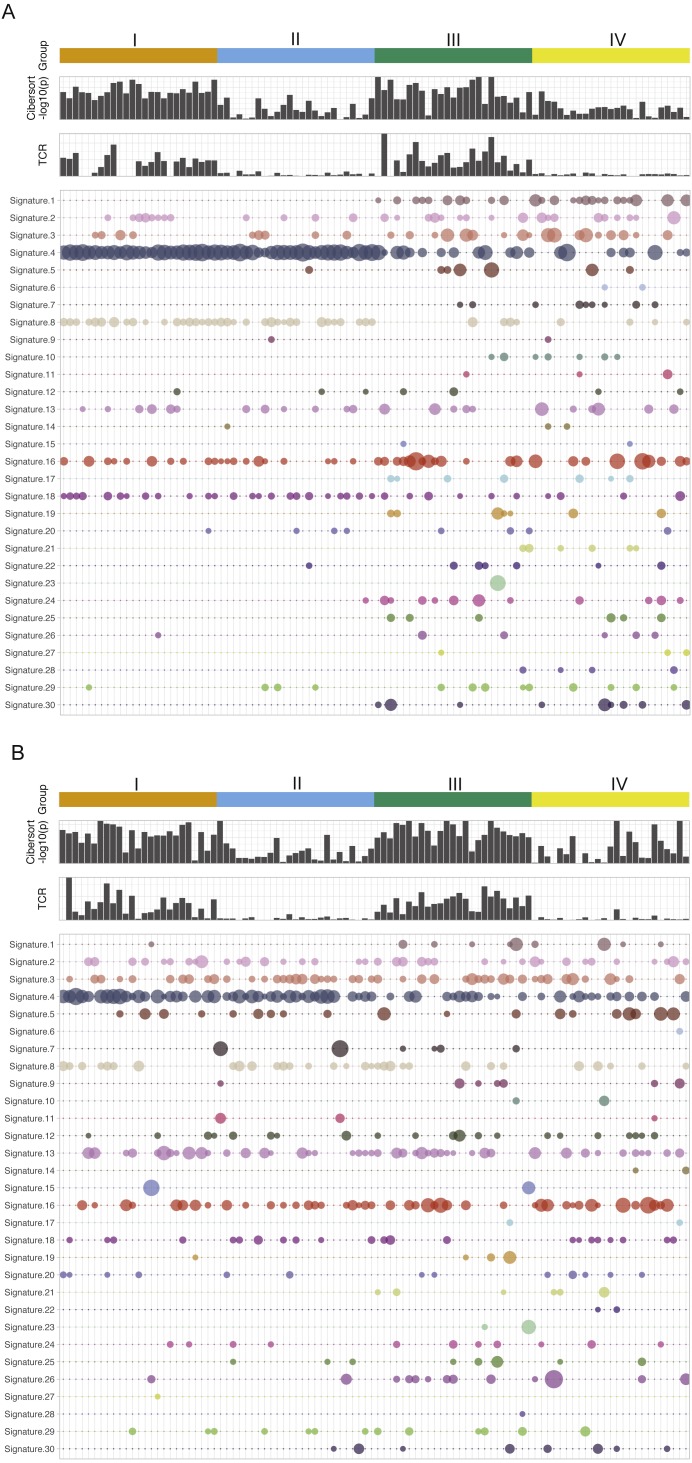
The mutational signatures and immune scores of LUAD (A) and LUSC (B). The mutational spectrum, the estimated TCR score, and CIBERSORT *p*-value (−log10 scale) of four patient groups in the TCGA LUAD and LUSC cohorts: I, high mutation load and high CYT, II, high mutation load and low CYT, III, low mutation load and high CYT, and IV, low mutation load and low CYT. Each column represents one patient sample. Thirty mutational signatures cataloged at COSMIC are illustrated in the plot (each with a different color). The size of a circle in the mutational plot represents the calculated mutation percentage in one patient sample.

Figure 1 is the immune and mutation landscape of stratified TCGA lung cancer samples. There are three main differences in mutational signatures when comparing groups of high mutation load (groups I and II) and low mutation load (groups III and IV) in the LUAD cohort. (1) As expected, mutational landscape of high-mutation groups is clearly driven by signature 4, which is associated with smoking and tobacco mutagens; Signature 4 is significantly enriched in first two subgroups, with a combined median value of 0.55 in groups I and II vs. 0 in groups III and IV. (2) Low-mutation group patients showed more diverse mutational landscape with mutational components well distributed across all 30 types, while the high-mutation group concentrates on few signatures such as 4, 13, 16 and 18. The signature diversity disparity of the four groups are further illustrated by box plots of standard deviations of signature scores in Fig. S3. (3) Besides smoking signature, it is obvious that signature 1 (likely associated with patient age) and signature 17 (unknown aetiology) are enriched in two low-mutation groups and signature 8 (unknown aetiology) is enriched in two high-mutation groups. A plot reflecting the extent of mutational signature enrichment among four groups are shown in Fig. S4. However, the three distinct patterns observed in LUAD groups become rather blurred in LUSC groups, except the smoking signatures—with a median signature value of 0.31 vs 0.032 in mutation-high and mutation-low groups. Interestingly, we also found that the mutational signature related to smoking is better correlated with the self-reported smoking status in LUAD than in LUSC, probably because those LUSC patients are more widely affected by tobacco. Most signatures such as signatures 2, 3 and 5 are more equally distributed across high land low mutation groups in LUSC compared to LUAD. The mutations of few LUSC patients are even dominated by unexpected signatures such as signature 7 (associated with ultraviolet light exposure) and signature 15 (more likely to be associated with small cell lung cancer). From LUAD samples, we found that the patients from CYT-high groups have much smaller p-values derived from CIBERSORT, which test the null hypothesis that none of the cells that comprise the signature matrix are presented a sample (Newman et al., 2015). This finding support a previous study in breast cancer (Ali et al., 2016), in which the author hypothesized that CIBERSORT p-values reflects the overall proportion of immune cells versus non-immune cells in a given sample. Furthermore, we also observe that the TCR clonality score derived from RNA-seq is much higher in cytolytic high groups. The immune pattern including TCR and CIBERSORT *p*-value in LUSC is consistent with the finding in LUAD samples. However, we do observe that few LUSC samples with very high immune cell infiltration were classified into group II and IV, and interestingly all of them have low TCR estimates. This finding indicates that TCR estimates correlate better with cytolytic activity and can serve as a better surrogate of CYT values and a potential candidate for immune response prediction in patients with more complicated squamous cell carcinoma. This analysis reveals no significance difference or pattern in terms of mutational signature distributions when comparing CYT-high and CYT-low groups in either LUAD or LUSC cohort. Correspondingly, current data dos not provide sufficient evidence to support the proposition that the smoking signature is associated with higher CYT in lung cancer.

**Figure 2:**
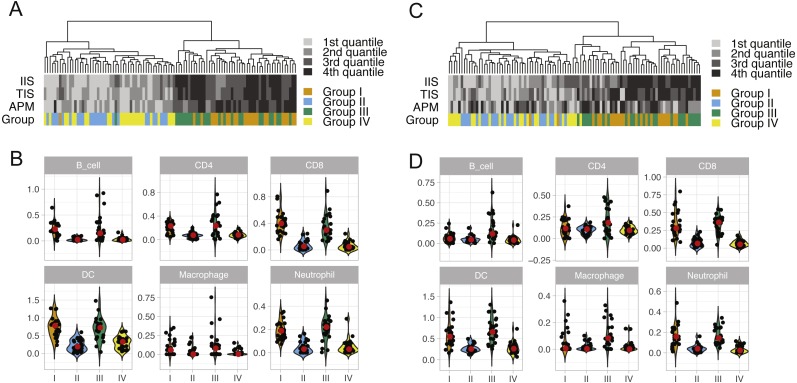
ssGSEA clustering analysis and the abundance of six immune cells in LUAD (A and B) and in LUSC (C and D) across four patient groups. The clustering analysis of lung cancer patients in four groups (A and C) were performed based on scores of 24 immune cell types calculated from the single sample gene set enrichment analysis. The overall immune infiltration score (IIS), T cell infiltration score (TIS) and antigen presenting machinery (APM) of each sample from ssGSEA are also shown in the figure, with a darker color bar indicating a higher score. The abundance of six major immune cell types in four lung cancer patient groups is illustrated using violin plots (which are a hybrid of boxplots and density plots) in (B) and (D).

We performed unsupervised clustering based on ssGSEA scores of 24 immune cell types of each lung cancer sample (Fig. S2). This analysis reveals two distinct clusters that separate cytolytic-high and cytolytic-low groups, as shown in Figs. 2A and 2C. In LUAD, except for one patient, all cases in groups I and III were classified into the second major cluster. In LUSC, except for two patients from group I, all cases in groups I and III were classified into the second major cluster. Patients in this cluster had a significantly higher ssGSEA score of cytotoxic cells and T cells, as well as slightly higher scores of Neutrophils, dendritic cells and B cells. In LUAD, the second cluster also showed significantly higher overall immune infiltration score (IIS), T cell infiltration score (TIS) and antigen presenting machinery (APM) score as defined previously. However, only IIS and TIS scores are consistently higher in the second cluster of LUSC patients. The clustering analysis based on ssGSEA did not reveal any apparent grouping of patients in terms of mutation burden. Next, we focused on the comparison of infiltration scores for six major immune cells (Li et al., 2016b) among four groups. In LUAD (Fig. 2B), all the six cell types showed a higher abundance in groups I and III (cytolytic-high groups). In LUSC (Fig. 2D), only CD8, dendritic cells and Netruophils had apparent higher scores in cytolytic-high groups, with only group III having higher scores of B cells and Macrophage. Overall, the LUAD cohort showed higher infiltration than the LUSC cohort in their cytolytic-high groups. Because the cell deconvolution scores from TIMER are relative abundance, they cannot be used in our application to infer whether LUSC had a higher diversity of cell types as shown in the figures. These results suggest that CYT value is sufficient to distinguish immune infiltration groups while both CYT value and mutation status are important in predicting immune infiltrates in LUSC.

## Discussion

In this study, we conducted an in-depth immune and mutation analysis of all non-small cell lung cancer samples in the TCGA database. This analysis is timely because the full mutation annotation information for lung cancer, especially LUSC, only became publicly available very recently. We focus on the comprehensive comparison among patient stratification groups defined by their mutation and cytolytic activity status. We did not find an apparent association between mutation burden and CYT values, which were calculated based on the expression of GZMA and PRF1. Therefore, a total of 100 patients with extreme CYT and mutation burden from each of LUAD and LUSC cohort were selected for further downstream analysis. Patients from these groups did not show any survival differences based on current therapies, but their distinct immune and genomic characteristics might help identify patient groups and novel signatures for a more efficient immunotherapy, as well as the development of combined treatments. Thus, our first hypothesis was that these immunologically and mutational “cold” and “hot” tumors can have different immunotherapy responses, and the interactions between these two signatures may exist. Our second hypothesis was that the composition and diversity of tumor mutational signatures might provide a more accurate predictor than the total mutation burden. However, the results from decomposing mutational signatures showed that the mutational diversity is totally confounded with total mutation load in the tested samples, suggesting that the mutation reconstruction analysis may not be able to provide additional insights for lung cancer immunotherapy. As expected, the mutation-high patient groups in LUAD and LUSC were dominated by the signature related to smoking, while two mutation-low groups showed more diversity in mutation types. A signature with unknown aetiology (signature 8) was also clearly enriched in LUAD mutation-high groups, but not in LUSC. It is also reasonable to observe that the signature related to age (signature 1) was enriched in the mutation-low LUAD patients. Overall, LUSC samples showed much more complicated mutational composition than LUAD. Nevertheless, both cancer subtypes showed very similar patterns when we compare immune and genomic characteristics of cytolytic-high and cytolytic-low patient groups. We observed that the CYT values are strongly associated with higher total immune infiltration scores, T cell infiltration scores and TCR clonality scores. There were no apparent differences in mutational signatures when we compare different CYT groups. However, we did find that the immunologically hot and mutation-high patients tend to have higher immune scores compared to other patient groups in LUSC. This finding supports the view that both mutation load and CYT can be important predictors of immune response in LUSC. Based on the frequency table of all immune and genomic characteristics, it remains inconclusive, or even conflicting, regarding whether smoking signature and higher immune infiltrates–or their interactions–will make patient more responsive to immunotherapy.

It is interesting to compare our findings with previous reports on immune cytolytic activity analysis. A recent stratification analysis of pancreatic cancer (Balli et al., 2017) also found that high cytolytic activity did not correlate with increased mutation or neoepitope load, but was associated with established subtypes and distinct mutational events. Of note, they also found that LUAD showed a strong correlation between the total numbers of mutations, predicted MHC class I neoepitope load in cytolytic-high tumors. As shown in Fig. S5, we also found that tumors with high CYT exhibited increase expression of immune checkpoint genes such as CD274 (PDL1), TIGIT, HAVCR2 (TIM3) and CTLA4. Rooney et al. (2015) found that LUAD from ever-smokers had significantly higher CYT. However, we argue that this result should be interpreted with caution because of the strong confounding from the total mutation load. Similarly, any observed association between CYT and mutation events should be carefully examined due to the limited sample size and unknown factors that affect mutation patterns in tumors.

Taken together, we have demonstrated that the utility of integrating both genomic and immune landscape for a better understanding of immune response in lung cancer. Just like the UV signature in melanoma, both mutational and immune profiles of tumors are greatly influenced by mutational signature related to smoking. In the patient groups that are less dominated by the smoking and tobacco event, such as LUSC and the two mutation-low groups in LUAD, we speculate that the reconstruction of mutational compositions plays a more important role in searching for predictive factors underlying therapy response and clinical outcomes. Unfortunately, all the existing studies, including ours, are greatly limited by the lack of samples with immunotherapy response information. To date, TCGA is still the largest public resource of immune and genomic data in cancer. We have observed a substantial heterogeneity in both immune and genomic landscape of lung cancer tumor samples. The LUSC subtype has showed a higher extent of heterogeneity, probably due to the lack of specific driver factors. The tumor infiltration spectrum observed in this study may not represent the general patient population because the TCGA cohorts were biased towards selecting patient samples with higher tumor purity. Thus, it is important to validate our results using intendent lung cancer dataset in the future. Further, we will need to further validate the above hypotheses and potential predictive signatures on coming dataset with the response data to immunotherapies in non-small lung cancer such as anti-PD-1 (Rizvi et al., 2015). On the basis of our results, we expect that the most reproducible immune predictive signatures for lung cancer shall combine both mutational and gene expression signatures. Our findings also suggest that it will be necessary to look beyond existing molecular decomposition tools and standard tumor/immune scores and to include features from different sources, such as proteomics, metabolomics and mutations in germline.

## Conclusions

In conclusion, combining tumor genomic and immunology profiling will lend valuable insights into the future development of immunotherapy for non-small cell lung cancer. We expect that the proposed analytical strategy will yield more informative results and potential prognostic signatures when more response data will become available as the immune checkpoint inhibitor therapy becomes more widely adopted in the area.

##  Supplemental Information

10.7717/peerj.4546/supp-1 Supplemental Information 1 Supplementary Figures Figure S1. The scatter plots of mutation load and CYT values in LUAD (A) and LUSC (B).
Figure S2. Heatmap and clustering analysis of relative cell infiltrates based on estimates from ssGSEA for LUAD (A) and LUSC (B).
Figure S3. Standard deviations of mutational signatures (per patient) in four groups.
Figure S4. Enrichment plot of 30 mutational signatures in LUAD and LUSC cohorts.
Figure S5. Correlation of CYT values and immune checkpoint genes (all TCGA lung cancer samples).

10.7717/peerj.4546/supp-2 Table S1 TCGA Patient ID TCGA Patient ID, CYT values and mutation load in four stratified patient groups.

## Additional Information and Declarations

### Data Availability

The following information was supplied regarding data availability: Molecular and Clinical data of TCGA lung cancer samples were downloaded from the cBio Cancer Genomics Portal (http://www.cbioportal.org/), Broad Firehose website (https://gdac.broadinstitute.org/), and from Genomic Data Commons Data Portal (https://portal.gdc.cancer.gov/). The TCGA patient ID of four groups defined in this paper is provided in Table S1.

## References

[ref-1] Alexandrov LB, Jones PH, Wedge DC, Sale JE, Campbell PJ, Nik-Zainal S, Stratton MR (2015). Clock-like mutational processes in human somatic cells. Nature Genetics.

[ref-2] Alexandrov LB, Nik-Zainal S, Wedge DC, Aparicio SAJR, Behjati S, Biankin AV, Bignell GR, Bolli N, Borg A, Børresen-Dale A-L, Boyault S, Burkhardt B, Butler AP, Caldas C, Davies HR, Desmedt C, Eils R, Eyfjörd JE, Foekens JA, Greaves M, Hosoda F, Hutter B, Ilicic T, Imbeaud S, Imielinski M, Jäger N, Jones DTW, Jones D, Knappskog S, Kool M, Lakhani SR, López-Otín C, Martin S, Munshi NC, Nakamura H, Northcott PA, Pajic M, Papaemmanuil E, Paradiso A, Pearson JV, Puente XS, Raine K, Ramakrishna M, Richardson AL, Richter J, Rosenstiel P, Schlesner M, Schumacher TN, Span PN, Teague JW, Totoki Y, Tutt ANJ, Valdés-Mas R, Van Buuren MM, Van’t Veer L, Vincent-Salomon A, Waddell N, Yates LR, Zucman-Rossi J, Andrew Futreal P, McDermott U, Lichter P, Meyerson M, Grimmond SM, Siebert R, Campo E, Shibata T, Pfister SM, Campbell PJ, Stratton MR, Australian Pancreatic Cancer Genome Initiative, ICGC Breast Cancer Consortium, ICGC MMML-Seq Consortium, ICGC PedBrain (2013). Signatures of mutational processes in human cancer. Nature.

[ref-3] Ali HR, Chlon L, Pharoah PDP, Markowetz F, Caldas C (2016). Patterns of immune infiltration in breast cancer and their clinical implications: a gene-expression-based retrospective study. PLOS Medicine.

[ref-4] Balli D, Rech AJ, Stanger BZ, Vonderheide RH (2017). Immune cytolytic activity stratifies molecular subsets of human pancreatic cancer. Clinical Cancer Research.

[ref-5] Charoentong P, Finotello F, Angelova M, Mayer C, Efremova M, Rieder D, Hackl H, Trajanoski Z (2017). Pan-cancer immunogenomic analyses reveal genotype-immunophenotype relationships and predictors of response to checkpoint blockade. Cell Reports.

[ref-6] Li B, Li T, Pignon J-C, Wang B, Wang J, Shukla SA, Dou R, Chen Q, Hodi FS, Choueiri TK (2016a). Landscape of tumor-infiltrating T cell repertoire of human cancers. Nature Genetics.

[ref-7] Li B, Severson E, Pignon J-C, Zhao H, Li T, Novak J, Jiang P, Shen H, Aster JC, Rodig S, Signoretti S, Liu JS, Liu XS (2016b). Comprehensive analyses of tumor immunity: implications for cancer immunotherapy. Genome Biology.

[ref-8] Newman AM, Liu CL, Green MR, Gentles AJ, Feng W, Xu Y, Hoang CD, Diehn M, Alizadeh AA (2015). Robust enumeration of cell subsets from tissue expression profiles. Nature Methods.

[ref-9] Patel SJ, Sanjana NE, Kishton RJ, Eidizadeh A, Vodnala SK, Cam M, Gartner JJ, Jia L, Steinberg SM, Yamamoto TN, Merchant AS, Mehta GU, Chichura A, Shalem O, Tran E, Eil R, Sukumar M, Guijarro EP, Day C-P, Robbins P, Feldman S, Merlino G, Zhang F, Restifo NP (2017). Identification of essential genes for cancer immunotherapy. Nature.

[ref-10] Rizvi NA, Hellmann MD, Snyder A, Kvistborg P, Makarov V, Havel JJ, Lee W, Yuan J, Wong P, Ho TS, Miller ML, Rekhtman N, Moreira AL, Ibrahim F, Bruggeman C, Gasmi B, Zappasodi R, Maeda Y, Sander C, Garon EB, Merghoub T, Wolchok JD, Schumacher TN, Chan TA (2015). Mutational landscape determines sensitivity to PD-1 blockade in non–small cell lung cancer. Science.

[ref-11] Rooney Michael S, Shukla Sachet A, Wu Catherine J, Getz G, Hacohen N (2015). Molecular and genetic properties of tumors associated with local immune cytolytic activity. Cell.

[ref-12] Rosenthal R, McGranahan N, Herrero J, Taylor BS, Swanton C (2016). deconstructSigs: delineating mutational processes in single tumors distinguishes DNA repair deficiencies and patterns of carcinoma evolution. Genome Biology.

[ref-13] Schumacher TN, Schreiber RD (2015). Neoantigens in cancer immunotherapy. Science.

[ref-14] Şenbabaoğlu Y, Gejman RS, Winer AG, Liu M, Van Allen EM, De Velasco G, Miao D, Ostrovnaya I, Drill E, Luna A, Weinhold N, Lee W, Manley BJ, Khalil DN, Kaffenberger SD, Chen Y, Danilova L, Voss MH, Coleman JA, Russo P, Reuter VE, Chan TA, Cheng EH, Scheinberg DA, Li MO, Choueiri TK, Hsieh JJ, Sander C, Hakimi AA (2016). Tumor immune microenvironment characterization in clear cell renal cell carcinoma identifies prognostic and immunotherapeutically relevant messenger RNA signatures. Genome Biology.

[ref-15] Snyder A, Makarov V, Merghoub T, Yuan J, Zaretsky JM, Desrichard A, Walsh LA, Postow MA, Wong P, Ho TS, Hollmann TJ, Bruggeman C, Kannan K, Li Y, Elipenahli C, Liu C, Harbison CT, Wang L, Ribas A, Wolchok JD, Chan TA (2014). Genetic basis for clinical response to CTLA-4 blockade in melanoma. New England Journal of Medicine.

[ref-16] Subramanian A, Tamayo P, Mootha VK, Mukherjee S, Ebert BL, Gillette MA, Paulovich A, Pomeroy SL, Golub TR, Lander ES, Mesirov JP (2005). Gene set enrichment analysis: a knowledge-based approach for interpreting genome-wide expression profiles. Proceedings of the National Academy of Sciences of the United States of America.

