# Supplementary material for: Tumor immunity landscape in non-small cell lung cancer
Source: PeerJ. 2018 Mar 23;6:e4546. doi: 10.7717/peerj.4546 (PMC5868477; doi:10.7717/peerj.4546)
Supplement: Supplemental Information 1 — Figure S1. The scatter plots of mutation load and CYT values in LUAD (A) and LUSC (B). Figure S2. Heatmap and clustering analysis of relative cell infiltrates based on estimates from ssGSEA for LUAD (A) and LUSC (B). Figure S3. Standard deviations of mutational signatures (per patient) in four groups. Figure S4. Enrichment plot of 30 mutational signatures in LUAD and LUSC cohorts. Figure S5. Correlation of CYT values and immune checkpoint genes (all TCGA lung cancer samples). [file peerj-06-4546-s001.pdf]

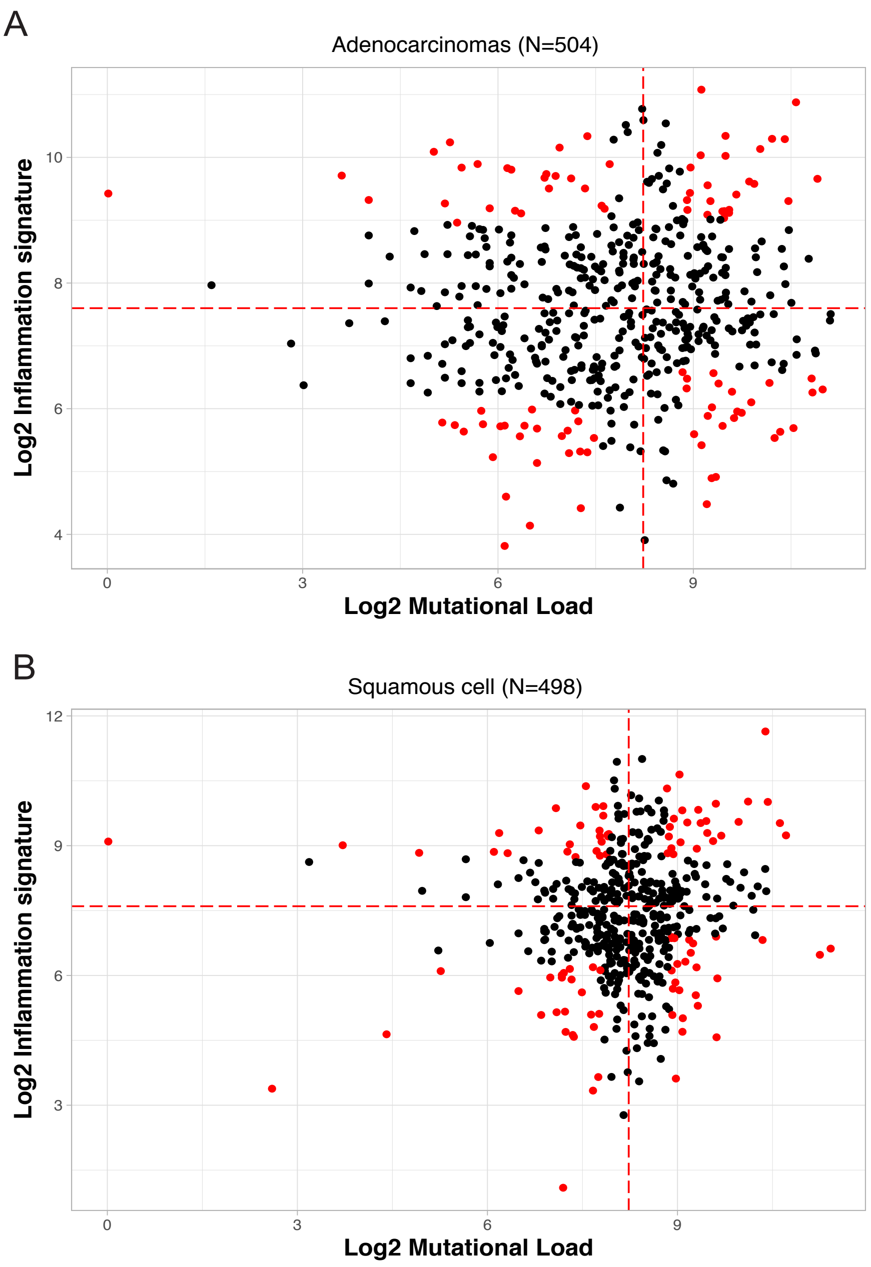

Supplementary Figure 1. The scatter plots of mutation load and inflammation signature (CYT values) in LUAD (A) and LUSC (B). The CYT values were calculated as log-average expression of *GZMA* and *PRF1*. (A) LUAD patients with extreme mutational load and CYT values were evenly selected from 4 groups (25 per group): group I with high mutation load (>450) and high CYT (log-average >9); group II with high mutational (>450) load and low CYT (log-average <6.57); group III with low mutational load (<210) and high CYT (log-average >8.95); group IV with low mutational load (<190) and low CYT (log-average <5.98). (B) LUSC patients with extreme mutational load and CYT values were evenly selected from 4 groups (25 per group): I with high mutation load (>450) and high CYT (log-average >8.7); II with high mutational (>450) load and low CYT (log-average <6.85); III with low mutational load (<240) and high CYT (log-average >8.7); IV with low mutational load (<219) and low CYT (log-average <6.3).

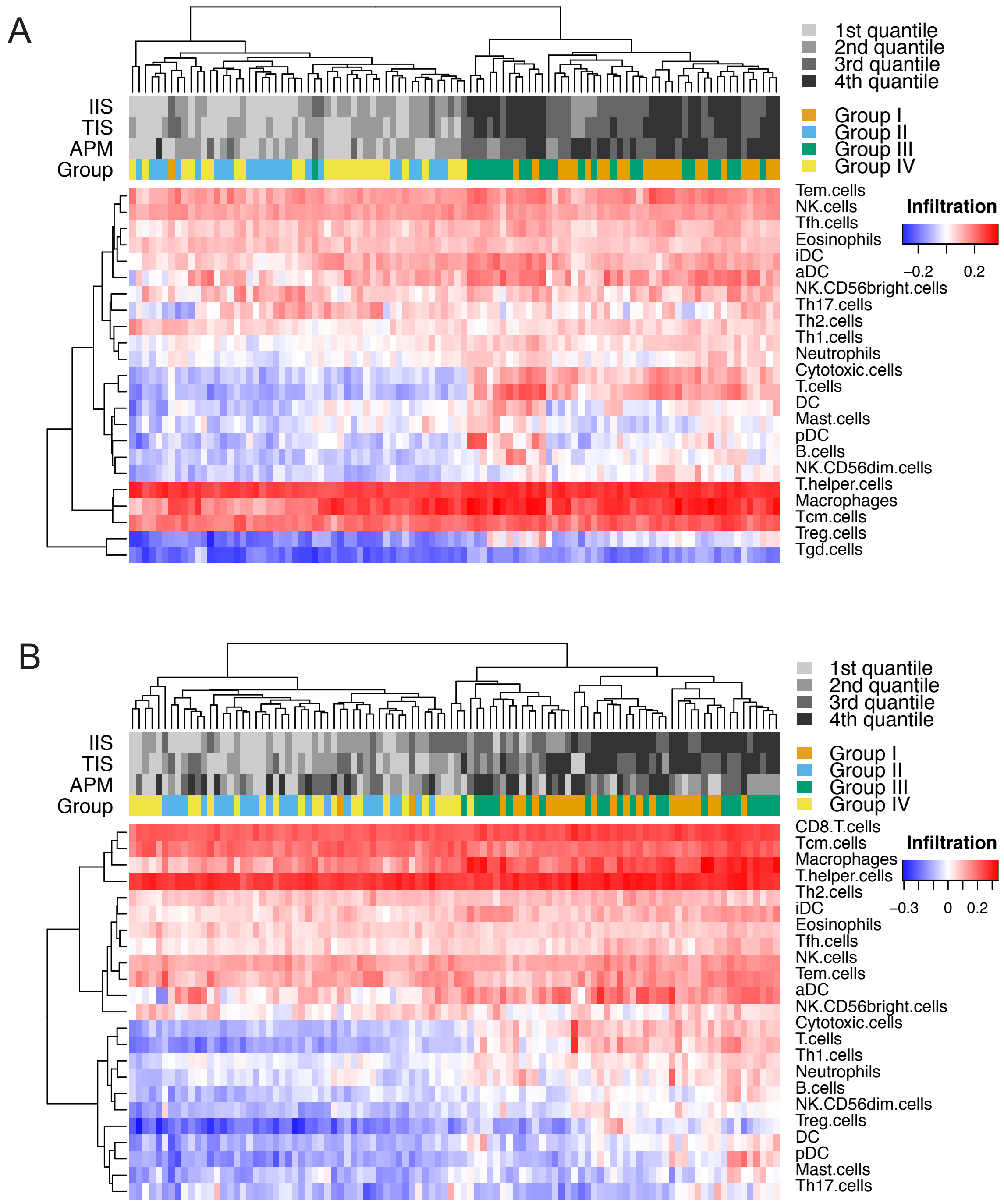

Supplementary Figure 2. Heatmap and clustering analysis of relative cell infiltrates based on estimates from ssGSEA for LUAD (A) and LUSC (B).

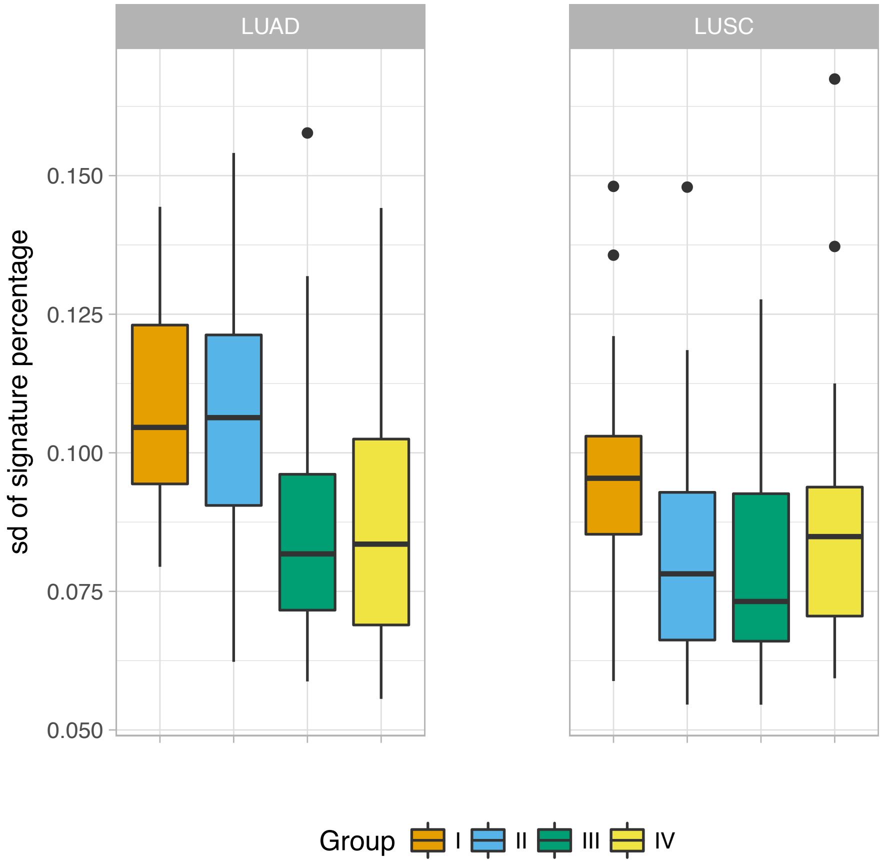

Supplementary Figure 3. Standard deviations of mutational signatures (per patient) in four groups. Larger standard deviations of mutational scores indicate smaller diversity. High-mutation group (I and II) patients showed less diversity of mutational landscape in LUAD, while only group I in LUSC samples shower relatively lower diversity.

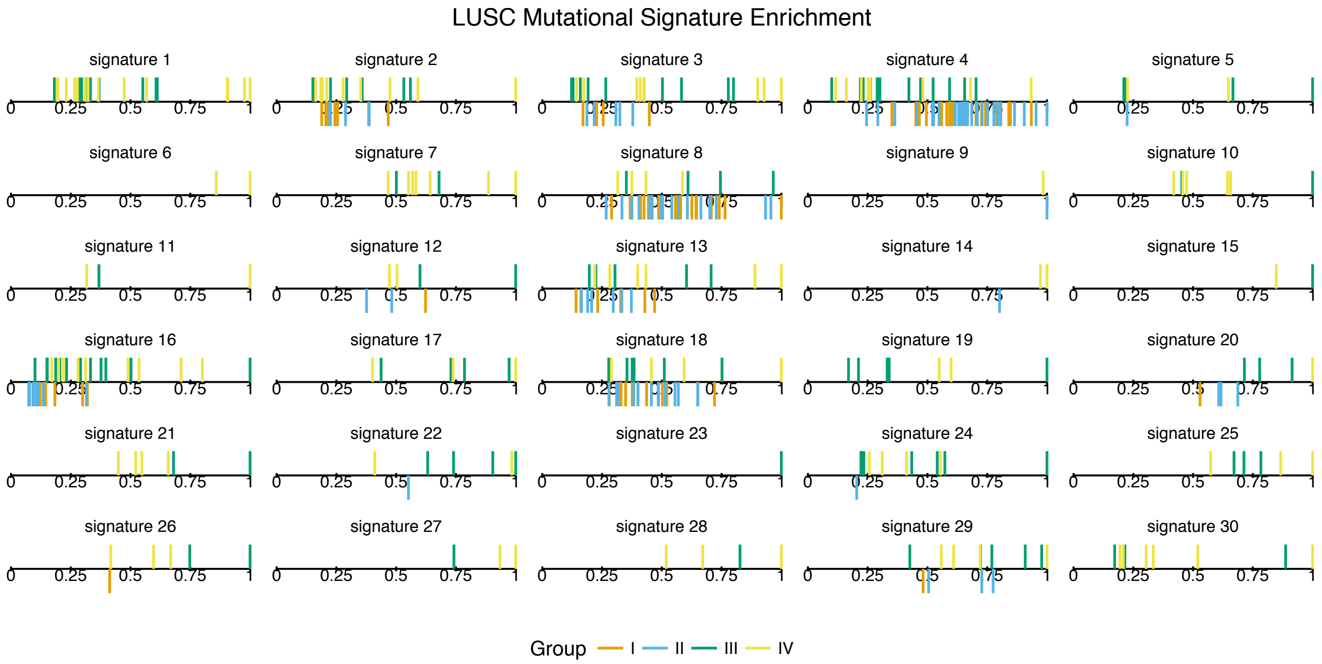

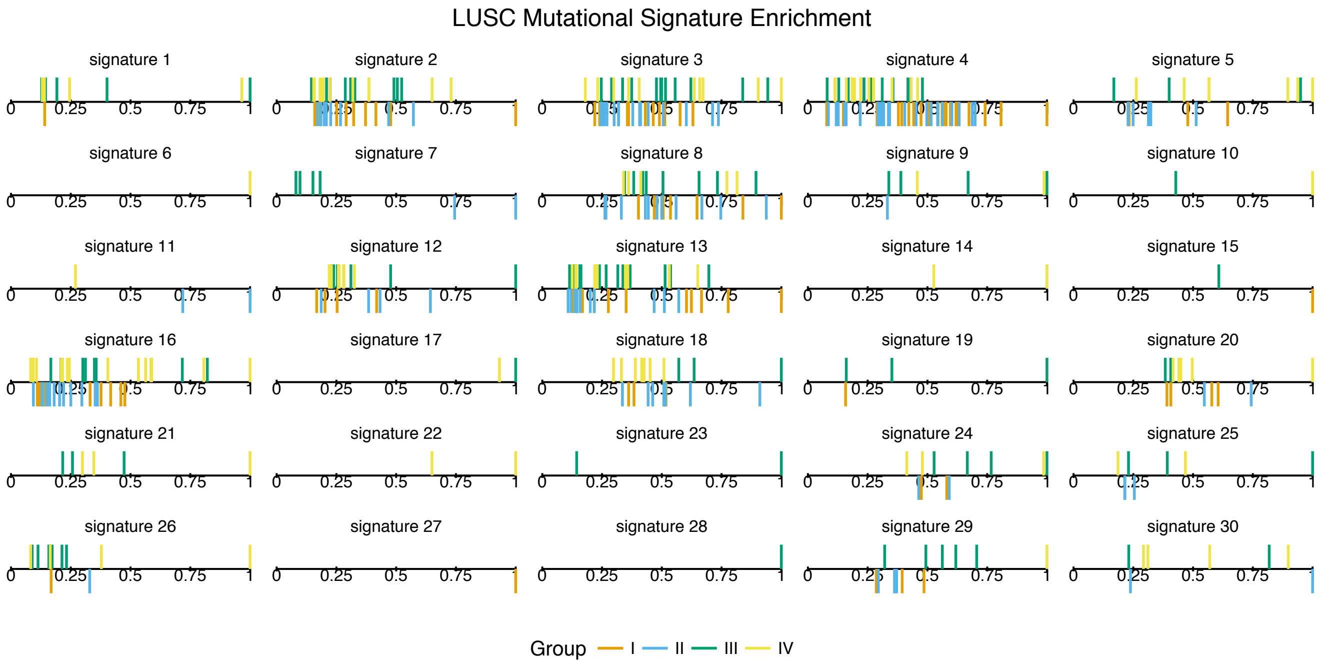

Supplementary Figure 4. Enrichment plot of 30 mutational signatures in LUAD and LUSC cohorts. The X axis are the scaled signature score (divided by the maximum value across all samples). The bars above the axis are patient values from low mutation groups (III and IV) and the bars below the axis are from the high mutation groups (I and II).

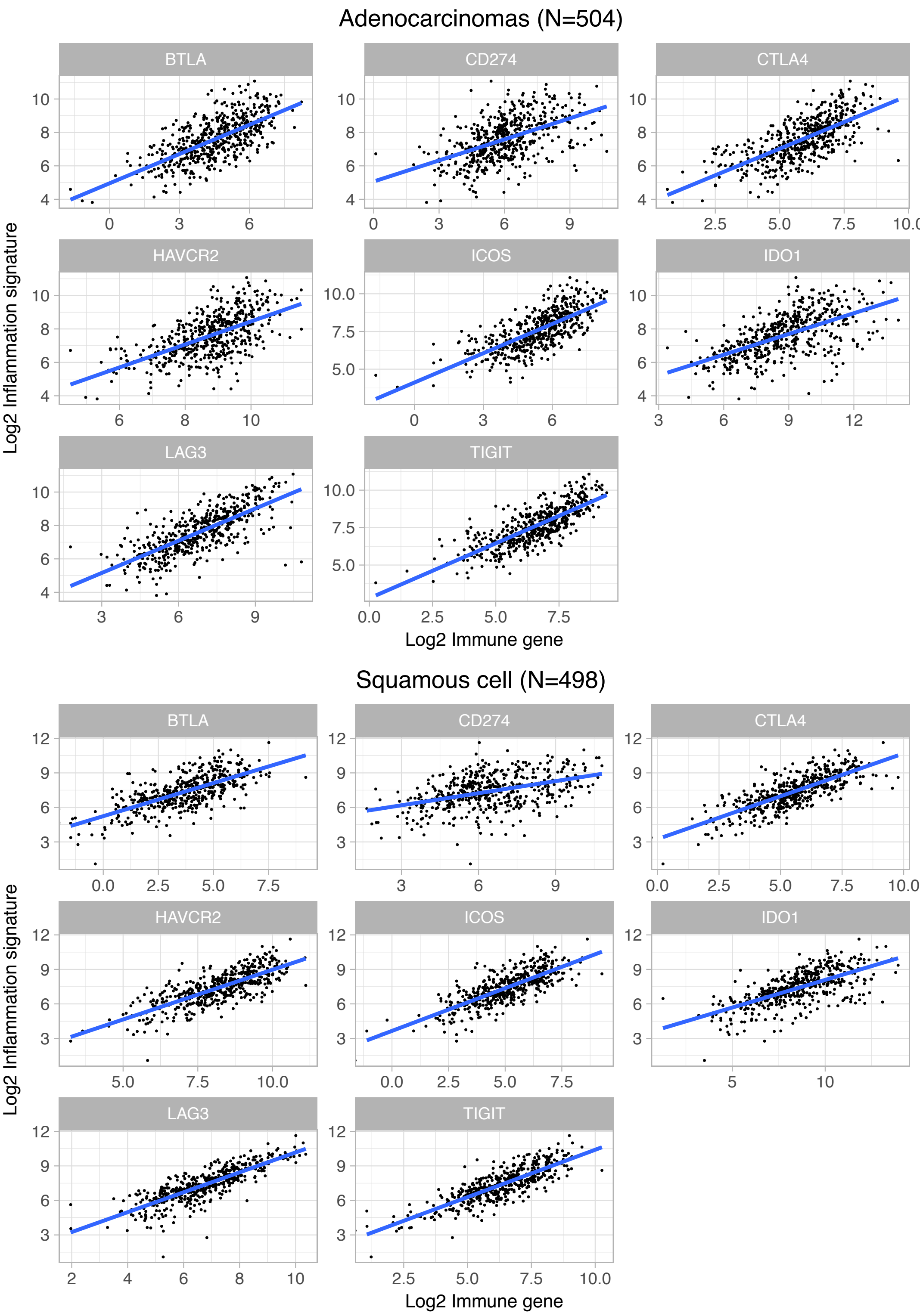

Supplementary Figure 5. Correlation of CYT values and immune checkpoint genes (all TCGA lung cancer samples).
